# Managing dental caries in children in Turkey - a discussion paper

**DOI:** 10.1186/1472-6831-9-32

**Published:** 2009-11-25

**Authors:** Asli Topaloglu-Ak, Ece Eden, Jo E Frencken

**Affiliations:** 1 Department of Paediatric Dentistry, Ege University, Bornova, Izmir, 35100, Turkey; 2 Nijmegen International Centre for Oral Health, Radboud University Nijmegen Medical Centre, College of Dental Sciences, Nijmegen, the Netherlands

## Abstract

**Background:**

This paper describes the oral healthcare system and disease situation amongst children in Turkey. Considering the high prevalence and severity of dental caries, a proposal for improvement of oral health in this population group is formulated.

**Discussion:**

A virtual absence of palliative, preventive and restorative care characterises juvenile oral healthcare in Turkey. Consequently, carious cavities remain untreated, which may lead to pain, discomfort and functional limitation and, further, may impact negatively upon general health and cognitive development. As a first step to controlling dental caries, a national health programme including promotional, preventive and minimal intervention approaches for managing dental caries is proposed. The pros and cons of community-oriented caries-preventive measures are discussed. Daily tooth brushing with fluoridated toothpaste at home, in mother- and child-care centres, kindergartens, and schools is highlighted.

**Summary:**

The dental profession, government, university officials and other stakeholders need to meet and determine how best the oral health of children in Turkey can be improved. The present proposed plan is considered a starting point.

## Background

Worldwide, the contribution of dental caries to the burden of oral diseases is about 10 times higher than that of periodontal disease, the other common oral condition [1]. Owing to its globally high prevalence, dental caries in children has been described as a 'pandemic' disease characterised by a high percentage of untreated carious cavities causing pain, discomfort and functional limitations [2]. Untreated carious cavities, furthermore, have a significant impact on the general health of children and on the social and economic wellbeing of communities [3] and are more common in developing, than in developed countries [4]. In the latter, the low prevalence of dental caries is predominantly due to children's intensive use of promoted preventive oral health measures, supported by appropriate curative care provided by an umbrella of accessible clinics and affordable health insurance. In contrast, most developing countries and those in transition lack the resources and infra-structure required to provide children with the necessary care and attention.

Turkey is a country in transition, whose economy has grown tremendously during the last decade. Although the proportion of the national budget allocated to healthcare has been increased, however, there are signs that the juvenile population suffers from the consequences of dental caries [5].

This paper aims to contribute to the discussion on how best to improve the management of dental caries in children in Turkey. It begins with a situation analysis of the oral health infra-structure, disease levels and utilization of oral care, continues with a discussion of possible solutions and concludes with some recommendations.

## Discussion

### Oral healthcare infrastructure

Turkey is an upper-middle-income country with a population of about 72 million people, 38% of whom lived in rural areas. Those under the age of 15 constitute 28% of the population [6]. It has more than 22,000 active dentists but no institutions for training auxiliary dental personnel. Almost 1000 dentists graduate from the country's 20 dental schools annually [6]. In 2007, 61% of the dentists worked in private practices, 24% in government hospitals, 9% in university hospitals and 6% in other institutions [6]. Most newly graduated dentists find employment in private practices, which are predominantly found in urban areas but are unevenly distributed over the country's 81 cities [6]. Of the total dentist population, 20% practise in İstanbul, which has a dentist-population ratio of 1:1978, whereas the highest dentist-population ratio of 1:1438 is found in the capital city, Ankara, where 5% of the dentists reside. The city of Şanlıurfa has the lowest dentist-to-population ratio (1:12,980). The ratio in rural areas is even lower. The average dentist-population ratio in Turkey is 1: 3209 in 2008, which is much lower than in most Western European countries [6].

Oral healthcare, like all other health services, is mainly provided by the Ministry of Health, SSK (Social Insurance Organization), universities, the Ministry of Defence, private practitioners and paramedical professionals. The number of private hospitals is low whereas not all of them are providing oral health care. The organisation of health service delivery needs to be improved.

### Oral healthcare financing

For a long time there were three main sources of healthcare financing in Turkey: the state, social security institutions and direct payment by the patient. During the last decades health insurance companies have emerged, offering services throughout the country. This has resulted in the private health insurance sector's having become the country's most fast-developing insurance branch. However, oral health treatments have rarely been included in the package and the patient pays the private dental practitioner directly.

Deficiencies of the health insurance systems, with results such as unequal and unaffordable use of oral health services, led politicians to call for the development of a new health insurance system for all. The national health insurance system was introduced in 2008 and covers oral healthcare. It aims at decreasing the cost of health services and encouraging preventive health practices. As part of the introduced changes, some groups in the society are given special preference: for example in oral health, children aged between 5 and 15 years are entitled to apply to any of the institutions and the private sector for orthodontic treatments, restoration of teeth and root canal therapy on first and second permanent molars, on demand. People ineligible for participation in the national health insurance but accepted as very poor are entitled to free oral healthcare, with the exception of orthodontic and prosthodontic treatment. However, the introduction of the new insurance system has increased the demand for dental services in state hospitals and clinics, resulting in long waiting queues. Consequently, the rural population and the urban poor experience difficulty in obtaining dental services when they need them.

### Utilization of dental services

It has been reported that the ordinary Turkish person does not perceive oral health as important and considers it to be of low priority [7]. An adult survey of residents from 9 provinces revealed that only 40% had visited dental facilities within the previous year [8]. The predominant reason for using the services was the occurrence of a dental problem, less than 2% of adults having visited the dental facilities for a routine check-up [8]. The attendance pattern among children was similar. The 2004 national oral health survey revealed that 18% of 5-year-olds and 59% of 12- and 15-year-olds had visited a dentist [5]. The main reason among 5-year-olds was that they required a check-up, while among 12-year olds (55%) and 15-year olds (46%) it was the need for tooth extraction. In these ages, the predominant motivation for attending dental clinics was pain-driven.

It can be concluded that utilization of oral health services among children and adolescents is low to medium, and irregular. Seeking relief from pain/toothache is the main reason given for visiting the dentist. The routine of going for regular check-ups has not been established.

Cross-cultural studies have revealed that dental attitudes, knowledge and behaviour are dependant upon cultural beliefs and economic factors. The family structure in Turkey is characterized by high parental control, protectiveness and involvement with children [9]. Very few studies have been conducted on behavioural and cultural aspects related to oral health in Turkey. It is generally accepted that dental attendance rates increase with an increase in the educational status of the people [8] and that mothers' education levels play an important role in their children's oral health. In Turkey women's primary role is to care for the family and raise children. Their levels of dental anxiety correlate positively with those of their children, so a child may develop a high level of anxiety if the dental anxiety of the mother is high [10]. It is reported that women with more than 11 years of education have fewer children than those with less and achieve higher socioeconomic status [11] and that a higher socio-economic maternal profile positively affects the dental visit frequency, dental anxiety and oral health of the children [10,11].

Turkey has not yet developed a system in which routinely regular dental visits are the accepted norm. In addition, oral health culture has not been developed either. It appears, therefore, that the population needs to be educated about the advantages of regularly visiting a dentist. For the dental caries prevalence among the youth to be reduced and their oral health to be improved, responsible policymakers would need to develop and implement appropriate oral health promotion and care programmes for use in mother-and-child health centres, day-care centres and primary schools. Links with the private sector, which provides the lion's share of oral care in the country, should also be established. Studies evaluating the appropriateness and effectiveness of the oral care delivery systems in the country are not currently available.

### Oral health status of children

Few oral-health-related epidemiological surveys covering children have been carried out. Most of these were conducted in cities, in dental schools at universities, and covered low numbers of participants [12-14]. Therefore, the general information regarding the oral health status of children in Turkey originates from the two national surveys of 1988 [15] and 2004 [5]. The latter one was carried out by the Ministry of Health in cooperation with Hacettepe University. In both surveys WHO criteria [16,17] were used in diagnosing caries respectively. The caries prevalence and caries experience (dmft) in 6-year-olds in 1988 were 84% and 4.4, and in 5-year-olds in 2004 they were 70% and 3.7, respectively. Both surveys found hardly any restorations in these age groups. The caries prevalence and caries experience (DMFT) of 12-year-olds were 84% and 2.7 in 1988, and 61% and 1.9 in 2004. The care index of the 12-year-olds was low: 11% in 1988 and 5% in 2004. Both studies show a high prevalence of dental caries and a high caries experience among 5-6-year-olds and 12-year-olds. Only 1% of 5-6-year-olds had had decayed teeth filled.

### Characteristics of caries control in Turkey

Dental services in Turkey are mainly technically oriented. A firm belief persists that caries control is achieved through technical perfection in restorative care. For many, caries management is still based on the currently outdated paradigm that carious lesions can be treated through mechanical methods only. Reference is made to the principles of GV Black's cavity design and use of the 'extension for prevention' concept. General dental practitioners in Turkey do not frequently apply even the technically preventive measure of sealing fissures, owing to a lack of interest and insufficient knowledge [18]. The traditional restorative approach to managing dental caries has been deeply integrated into the legislative and remuneration systems, the dental school curricula and public knowledge. Some dental schools have recently embraced the modern cariology concepts but implementation of these in practice is seriously hampered by the restorative-oriented insurance system. They teach sealant and fluoride application. It appears that not only do dental schools need to change the emphasis in their curricula, from traditional restorative care towards applied preventive and modern restorative care concepts, but education should also be further updated for the older graduates.

### Oral health education and promotion

Oral health education (OHE) aimed at improving oral health through the acquisition of knowledge, eventually leading to motivation and finally, to behavioural change according to the health belief model, has for decades been considered the panacea that would provide people with better oral health. OHE studies have been performed in many countries [19,20] including Turkey [21-23]. However, accumulating evidence reveals that focusing on individual behaviour, without giving attention to the socio-economic, cultural and ecological situations in which people live, does not lead to improved oral health [24]. It appears that individuals do not easily change their behaviour, because it is mainly determined by the environment [25,26].

As sustainable benefits of OHE are not apparent and comprehensive and OHE programmes are expensive in terms of finance and human resources, the health authorities in Turkey, as part of their oral health promotion strategies, need to focus on pruning the OHE programmes in order to provide essential information only.

### Community-oriented evidence-based caries-preventive measures

In principle there are three ways to control carious lesion development: by weakening promoting factors (e.g. sugar consumption), by reinforcing protective factors (e.g. fluoride) or by a combination of both. It is postulated that behavioural changes regarding dietary habits at a national level may be achievable through approaches similar to those used in reducing smoking: strong regulation of food labelling, restrictions on advertising of unhealthy food, taxation of unhealthy food, legislation to control unhealthy food, better accessibility to healthy food and a ban on the sales of unhealthy and sugary foods in and around schools.

The other determinant for caries control is fluoride. Application of fluoride in various forms has shown dramatic effects in reducing the prevalence and severity of carious lesions. The oldest community-based fluoride delivery system is water fluoridation. The carious-lesion-reducing effect of water fluoridation, however, diminishes considerably if fluoridated toothpaste is used on a daily basis [27]. Therefore, as no fluoridated drinking water is available in Turkey and as it has been shown that caries levels diminish when people brush their teeth daily with fluoride toothpaste, introducing expensive water fluoridation schemes is not considered the best way forward for controlling dental caries development in the country [28].

Another community-based fluoride measure is salt fluoridation. Early salt fluoridation trials, in Hungary between 1966 and 1976 [29] and in Columbia between 1964 and 1972 [30], reported a reduction in carious lesions in children when all salt for human consumption was fluoridated. However, the evidence-based anticaries effect of salt fluoridation still has to be ascertained [31]. Embarking on a country-wide introduction of salt fluoridation in Turkey would therefore, not be advisable.

Encouraging daily tooth brushing with fluoride toothpaste is also considered a community-based measure, but compliance is a prerequisite for its effectiveness. A systematic review of the effectiveness of fluoride toothpaste for prevention of dental caries in children examined 74 randomized controlled trials and found, on average, a 24% reduction in decayed, missing and filled tooth surfaces (DMFS) in the permanent dentition of children aged 6 to 16 years [32]. The effect of fluoride toothpaste increased with higher baseline levels of DMFS, higher fluoride concentration, higher frequency of use, and supervised brushing. No significant differences were found between the trials using sodium monofluorophosphate, stannous fluoride, sodium fluoride, and amine fluoride as the active ingredient.

The conclusion, reached after consideration of the pros and cons of the above- mentioned community-oriented caries preventive measures, is that brushing teeth daily with fluoride toothpaste appears to be the best way of reducing the development of caries lesions in children and should therefore be strongly promoted. Brushing teeth with fluoride toothpaste has the additional effect of being beneficial for periodontal tissues, which no other fluoride delivery method provides.

Another focus of attention for oral health authorities should be on the development of an advocacy process and of policies promoting twice-daily brushing of teeth with fluoridated toothpaste [33].

### The need for a new strategy for managing dental caries in children

The new strategy should take the following into account:

• The low level of dental consciousness affecting children's dental health;

• The low level of access to and utilization of oral care;

• The high level of dental caries and the high percentage of untreated carious cavities in children;

• The consequences of untreated carious cavities in children;

• The absence of a coordinated national oral health promotion approach.

These issues warrant the formulation of a clear and feasible national oral health strategy focused on providing basic oral care for all children, using the resources available.

### Basic oral care for children

As has been argued above, the sequelae of untreated carious cavities have significant and often underestimated impacts on the wellbeing of children and communities. The reality is that carious lesions do not seriously affect children unless they cause pain, discomfort and functional limitations. There is no doubt that carious lesions should be treated preventively and if a cavity has developed, appropriate restorative care should be rendered. Traditional restorative dental treatment using rotary instruments is expensive. It has been estimated that 5% to 10% of public health spending in high-income countries is allocated to oral care, caries being the fourth-most expensive disease to treat [34]. Obviously, the costs exceed the financial resources available in low- and middle-income countries. What are the alternatives?

Extraction might be expected as a less expensive, easily administered treatment for pain relief in children. Studies have indicated that children with associated poor weight gain had an increase in weight after tooth-extraction [35]. Significant improvement in the children's eating preferences, quality of food eaten, social behaviour and sleeping habits was also reported by their parents after tooth extraction [36-38]. Therefore, extraction of painful primary teeth should be considered an adequate treatment for improving the general health of the child. Nevertheless, it should be taken into account that early loss of primary teeth such as incisors, causing aesthetic problems, or of molars, causing space loss in the dental arch, may lead to further problems. Extracting permanent teeth of children should be avoided as far as possible; particularly those teeth that affect appearance and speech (front teeth, including the first and second premolars).

The preventive and curative caries management approach of ART offers a suitable alternative to extraction of children's teeth. Initial caries lesions, which mainly occur in pits and fissures, can be treated with ART sealants, while caries cavities can be treated with ART restorations [39]. A meta-analysis revealed a 6-year survival rate of 72% for single-surface ART restorations, whereas the annual dentine lesion development for ART sealants was only 1% [40]. In view of the above, the following components of basic oral care for children are suggested:

a) Increasing availability of affordable and effective fluoride toothpaste;

b) Brushing of teeth with fluoride toothpaste at an early age, with parental help;

c) Participation in daily programmes of brushing teeth with fluoride toothpaste, to stimulate the habit of tooth-brushing in a community;

d) Restoration or extraction of carious cavities in primary teeth whenever needed;

e) Use of the ART approach as the first option for managing carious lesion development preventively and restoratively;

f) Adoption by private practitioners, of modern oral care concepts that render treatment in a child-friendly way and encourage children and parents to consult dental professionals;

g) Increasing of the oral health budget, by the government, to finance wider coverage of oral healthcare services for children. Representatives of the government, the dental association and the health insurance companies should reassess the dental insurance system and include remuneration for promotional and preventive oral health services.

These suggested components of a basic oral care programme for use in children in Turkey are further discussed below.

### Recommendations for achieving improved dental health among children

#### a) Increase the availability and usage of affordable and effective fluoride toothpaste

Fluoride toothpaste is available to a large section of the population. In recent years quality checks have been performed in a number of countries on the freely available ionisable fluoride in toothpaste. Results have shown that not all fluoride toothpastes contain sufficient amounts of freely available fluoride to be effective in controlling carious lesion development [41]. Setting up an independent system of quality control regarding the efficacy of fluoride toothpaste in Turkey would therefore be advisable. The recommendations specified in the WHO Basic Package of Oral Care could serve as a guide [42]. Affordability of fluoride toothpaste to consumers could be improved by stimulating local manufacturers to produce low-cost fluoride toothpaste and by introducing a government tax reduction or exemption.

#### b) Promote daily brushing of teeth with fluoride toothpaste

It will take some time for all Turkish children to get used to brushing their teeth with fluoride toothpaste daily. Therefore, this preventive measure should receive ample attention and needs to be vigorously advocated from the start. As parents will need to support children in brushing their teeth, an approach encouraging the whole population to brush their teeth twice daily should be the first priority. This advocacy process should be a concerted effort involving the dental profession, the government, the toothpaste industry, consumer associations and other stakeholders.

#### c) Stimulate the habit of daily tooth brushing with fluoride toothpaste in mother- and child- health centres, day-care centres and kindergartens

The introduction of programmes encouraging daily tooth-brushing with fluoride toothpaste in mother- and child-health centres, day-care centres and kindergartens is considered essential for promoting tooth-brushing behaviour in children and in the rest of the population. Mothers with small children are an important target group for the promotion of daily tooth-brushing with fluoride toothpaste. The rationale behind prioritizing oral healthcare for small children is that because they are at the beginning of their lives, the chance for preventing of disease and establishing lifelong healthy habits will be maximized [43]. The promotion of self-care and prevention at the earliest age possible has a dramatically beneficial effect on caries levels later in life [44]. Midwives and nurses regard themselves as important healthcare providers and are also highly respected by the mothers. These health personnel could play an important role in conveying the oral health message of the importance of brushing teeth daily with fluoride toothpaste at an early age [45]. In kindergartens and day-care centres, caretakers could supervise daily tooth-brushing with a pea-sized amount of fluoride toothpaste. The parents should be encouraged to ensure routine dental checkups, starting as early as possible. Nurses and caretakers of pre-school children should be trained to recognize toothache in young children, so as to be able to inform parents when a visit to a dentist is needed.

#### d) School health programmes

The 2007 WHO resolution on oral health includes an article entitled "develop and implement the promotion of oral health and prevention of oral diseases for preschool and schoolchildren as part of activities in health promotion schools". It explicitly calls on governments to engage actively in implementing the resolution. School-based OHE should be integrated into the broader frame of health promotion, according to the Ottawa Charter [46]. Since oral diseases share many risk factors inherent in other diseases [47], diet, hygiene, smoking, alcohol use, exercise and trauma are issues to be addressed by a school's health team. The Health Promotion Schools' Programme [48] emphasises a range of policies that create a healthy environment not only for students, but also for staff and the community. Such approaches have shown significant gains in a range of oral health outcomes, including caries and dento-facial injuries [49,50]. Their success is highly dependent on the participation of the community. Teachers, parents, and school and health authorities should participate in the planning, implementation and review processes of programmes like the Health Promotion Schools' Programme. In Indonesia and China daily kindergarten- and school-based programmes of tooth-brushing with fluoride toothpaste have shown a resultant 23-43% reduction in carious lesions [51,52].

Basic oral care for children requires that painful and infected primary teeth be treated; not neglected as they currently are. The ART approach of preventing and restoring initial and early cavitated caries lesions in primary and permanent teeth should be provided in primary schools. Studies in Turkey have shown that ART restorations can be performed adequately in schools [53] and are on a par with composite resin restorations [53,54]. These results indicate that expensive rotary equipment is not needed to provide preventive and restorative care as part of basic oral care for schoolchildren. MID is certainly less costly than traditional dentistry [55] and is provided in a child-friendly way [56,57]. However, the number of studies having assessed the effectiveness of OHP programmes is low.

#### e) Application of Minimal Intervention Dentistry (MID) by Dentists in Private Dental Clinics

Most dental care delivery to children and adults occurs in the consulting rooms of dental practices. If the oral health of children is to improve, dentists need to provide promotional, preventive and restorative care in a manner acceptable to the child. This implies, for example, that dentists should not necessarily use outdated traumatizing restorative techniques but should resort to modern approaches that are adequate and atraumatic for the child. Modern treatment approaches follow the principles of MID. This concept is based on understanding the biological approach to carious lesions and consists of accurate diagnosis, application of preventive measures, periodic monitoring and determination of the individual's risk status. When there is cavitation, an invasive operative approach is unavoidable. The focus must then be directed towards removing only infected dentine, while leaving affected dentine behind for remineralisation. Therefore, within MID, Black's standard cavity designs are redundant, as is the total removal of a defective failed restoration. The defective part of the restoration can be repaired instead.

The standard dental clinic is equipped with rotary machines. Studies have shown that many children are afraid of these [56,58]. Therefore, the use of the drill should be avoided as much as possible and less traumatic devices, such as hand instruments and chemomechanical caries removal gel, should be used instead. One suitable method is ART, which aims to treat carious cavities by using hand instruments. ART (which is in line with MID) can be used by dentists in modern dental clinics in Turkey, as it has been in other developed countries such as the UK [59], the USA [60] and the Netherlands [61], as an alternative to the conventional approach [54]. This less traumatic, child-friendly approach could also help to overcome dental anxiety in children, which is often triggered by traditional invasive operative treatments [56,62]. If dentists in private practice would adopt MID, universities stop teaching traditional restorative approaches only and offer postgraduate academic courses on MID, oral health for an increased number of children in Turkey could become a reality.

## Summmary

An effort has been made to improve the oral health of children in Turkey through describing the current oral health situation and through using evidence-based alternatives to the prevalent failing methods of providing oral care. The time is now ripe for the dental profession, the Turkish Dental Association, the government, university officials, and other stakeholders to discuss the current plan, make adjustments and agree on a set of guidelines that would make the plan work. One obvious aspect of a positive strategy would be a move from the current way in which oral health services are directed to children, towards preventive and promotional activities under the umbrella of Minimal Intervention Dentistry. The prevailing oral health strategies should be abandoned, as they have been shown to be not very beneficial to children.

## Competing interests

The authors declare that they have no competing interests.

## Authors' contributions

ATA, EE and JEF altogether collected the dental literature and wrote the manuscript. All authors read and approved the final manuscript.

## Pre-publication history

The pre-publication history for this paper can be accessed here:

http://www.biomedcentral.com/1472-6831/9/32/prepub
